# HIF1 and ID1 Interplay Confers Adaptive Survival to HIF1α-Inhibition

**DOI:** 10.3389/fcell.2021.724059

**Published:** 2021-11-08

**Authors:** Hao Geng, Hyun-Kyung Ko, Janet Pittsenbarger, Christopher T. Harvey, Changhui Xue, Qiong Liu, Sadie Wiens, Sushant K. Kachhap, Tomasz M. Beer, David Z. Qian

**Affiliations:** ^1^Prostate Cancer Research Program, OHSU Knight Cancer Institute, Oregon Health & Science University, Portland, OR, United States; ^2^Sidney Kimmel Comprehensive Cancer Center, Johns Hopkins University, Baltimore, MD, United States

**Keywords:** ID1, hypoxia, resistance, HIF1, targeted-treatment

## Abstract

Hypoxia is a universal pathological feature of solid tumors. Hypoxic tumor cells acquire metastatic and lethal phenotypes primarily through the activities of hypoxia-inducible factor 1 alpha (HIF1α). Therefore, HIF1α is considered as a promising therapeutic target. However, HIF inhibitors have not proven to be effective in clinical testing. The underlying mechanism is unclear. We report that oncogenic protein ID1 is upregulated in hypoxia by HIF1α shRNA or pharmacological inhibitors. In turn, ID1 supports tumor growth in hypoxia *in vitro* and in xenografts *in vivo*, conferring adaptive survival response and resistance. Mechanistically, ID1 proteins interfere HIF1-mediated gene transcription activation, thus ID1 protein degradation is accelerated by HIF1α-dependent mechanisms in hypoxia. Inhibitions of HIF1α rescues ID1, which compensates the loss of HIF1α by the upregulation of GLS2 and glutamine metabolism, thereby switching the metabolic dependency of HIF1α -inhibited cells from glucose to glutamine.

## Introduction

Solid tumor growth is inevitably accompanied by hypoxia, which activates the master transcription regulator – hypoxia inducible factors (HIFs) by increasing HIF1α and HIF2α (Harris, 2002; Giaccia et al., 2004; Bertout et al., 2008; Majmundar et al., 2010; Marignol et al., 2013). Although both HIFs promote oncogenesis, HIF1α appears to predominant in most types of human cancers (Sowter et al., 2003; Löfstedt et al., 2007; Ratcliffe, 2007; Dang et al., 2008; Keith et al., 2012). Clinically, an increase of HIF1α level is associated with advanced metastatic disease and/or patient mortality in almost all types of solid tumors (Semenza, 2007, 2009, 2010, 2012). Thus, HIF1α is considered as a promising therapeutic target, which in theory may improve disease outcome and patient survival (Giaccia et al., 2003; Semenza, 2003; Powis and Kirkpatrick, 2004). A growing number of HIF1α-inhibitory agents, including both chemical inhibitors (topotecan, PX-478, YC-1, 2-ME2, BAY87-2243, and digoxin) and antisense oligonucleotides (EZN-2968) have shown encouraging antitumor activities in blocking tumor growth and metastasis in multiple preclinical models (Semenza, 2012). However, the preclinical efficacy has not been recapitulated in clinical trials (clinicaltrials.gov). The mechanisms of resistance are not clear.

Inhibitor of DNA binding 1 (ID1) is an oncogenic protein, promoting cancer survival, proliferation, angiogenesis, and metastasis (Lyden et al., 1999; Perk et al., 2005; Gupta et al., 2007; Lasorella et al., 2014). ID1 protein has a helix-loop-helix domain (HLH), which negatively regulates the activity of HLH transcription factors (TF) by decreasing the TF/DNA-binding (Sun et al., 1991; Alani et al., 2001). Interestingly, HIF1α contains a HLH domain, which is essential for the assembly of HIF1-transcirptional complex and activity (Wang et al., 1995). Currently, the interactions between HIF1 and ID1 are unclear.

In this study, we found that ID1 protein is upregulated in response to HIF1α inhibition, and ID1 in turns supports HIF1-independent tumor growth in hypoxia and *in vivo*. Mechanistically, this stems from the negative interplay between HIF1α and ID1. The consequence is that ID1 is upregulated in response to HIF1α -targeted inhibition, which in turn compensates the loss of HIF1 by promoting metabolic adaptation *via* glutamine metabolisms.

## Results

### ID1 Protein Is Negatively Regulated by Hypoxia

**FIGURE 1:**
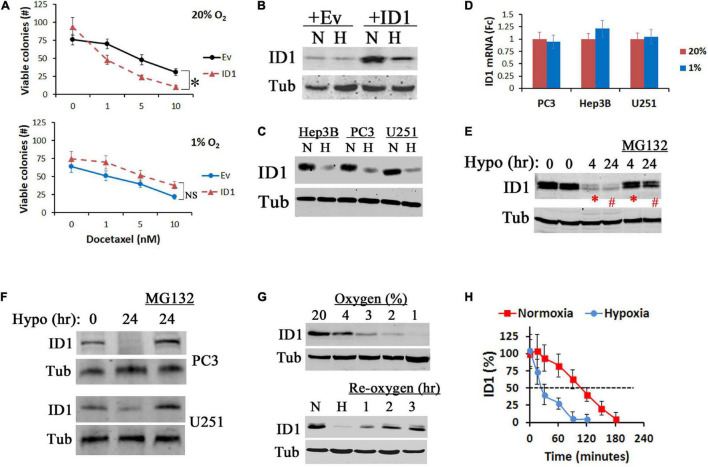
Hypoxia inhibits ID1 proteins. **(A)** LNCaP cells with stable ID1 (ID1) or empty vector (Ev) overexpression were treated with increasing dose of docetaxel in 20% or 1% O_2_. Cell viability was measured by colony formation assay. ^∗^*P* < 0.05, *t*-test, *n* = 3, mean and standard deviation (stdv). **(B)** LNCaP-Ev and LNCaP-ID1 cells were cultured in 20% (N) or 1% (H) O_2_ for 24 h. ID1 and tubulin (Tub) protein levels were determined by western blots. **(C,D)** Hep3B, PC3, and U251 cells were cultured in 20% (N) or 1% (H) O_2_ for 24 h. ID1 and tubulin were determined by western blots **(C)**. ID1 mRNA levels were determined by qRT-PCR with b-actin as control **(D)**. **(E)** Hep3B cells were cultured in 1% O_2_ for 4 and 24 h with/without proteasome inhibitor MG132. ID1 and tubulin were measured by western blots. **(F)** ID1 protein levels in PC3 and U251 cells after 24 h of normoxia, hypoxia, or hypoxia plus MG132. **(G)** ID1 protein levels after U251 cells were cultured with decreasing concentrations of O_2_ for 24 h (top). ID1 proteins after U251 cells cultured in 20% (N), 1% (H) O_2_ overnight, or 1% O_2_ overnight followed by re-oxygenation in 20% O_2_ for the indicated times (bottom). **(H)** U251 cells were cultured in 20% or 1% O_2_, and treated by cycloheximide to arrest protein synthesis. ID1 protein levels at the indicated times were determined by western blots.

Hypoxia reduces the efficacy of antitumor treatments. Previously, we found that stable overexpression of ID1 sensitizes prostate cancer cells to docetaxel chemotherapy by overwriting cell cycle checkpoints (Geng et al., 2010). In hypoxic condition, however, the ID1-based chemosensitization effect was significantly diminished (Figure 1A). In parallel, the plasmid-driven ID1 protein was reduced (Figure 1B). This hypoxia-dependent reduction of ID1 protein was present in a variety of cancer cell lines (prostate, liver, and brain) that express detectable levels of endogenous ID1 (Figure 1C). In all these cells, the reduction was at the protein level, as ID1 mRNA remained unchanged or slightly increased (Figure 1D), and ID1 protein was rescued by proteasome inhibitor MG132 (Figures 1E,F). To understand the hypoxia-induced protein degradation, we found that the inhibition of ID1 began when the oxygen concentration dropped below 4%, and re-oxygenation reinstated ID1 (Figure 1G). We next measured the kinetics of ID1 protein degradation with methods described by us previously (Geng et al., 2012). We found that hypoxia decreased ID1 protein half-life from ∼110 to ∼30 min (Figure 1H).

### HIF1α Accelerates ID1 Protein Degradation

**FIGURE 2:**
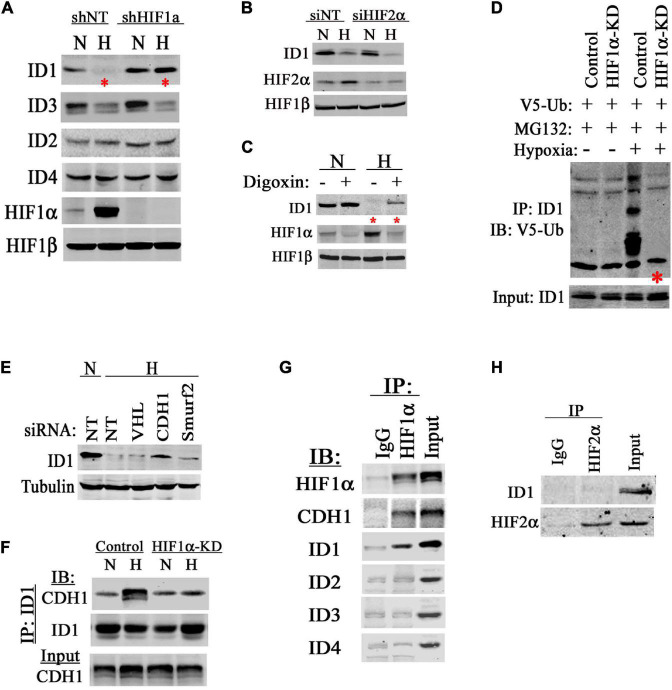
HIF1α accelerates ID1 protein degradations. **(A)** Western blots of ID1-ID4, HIF1α, and HIF1β in PC3 cells isogenic for HIF1α (shNT/shHIF1α) in 20% (N) or 1% (H) O_2_. **(B)** Western blots of ID1, HIF2α, and HIF1β in PC3 cells with siRNA silencing HIF2α in 20% (N) or 1% (H) O_2_. **(C)** Western blots of ID1, HIF1α, and HIF1β in PC3 cells being treated with HIF inhibitor Digoxin (Sigma) in 20% or 1% O_2_. **(D)** Hep3B cells with stable shRNA knockdown (KD) of HIF1α or non-targeting control (Control) were transfected with plasmid coding for V5-tagged ubiquitin. Cells were then treated with MG132 in 20% or 1% O_2_, ID1 protein was immunoprecipitated (IP) and immunoblotted (IB) for V5-ubiquitin modification. **(E)** Hep3B cells were treated with siRNA against VHL, APC/CDH1 (CDH1), or Smurf2 for 48 h. Afterward, cells were cultured in 20% (N) or 1% (H) O_2_ for 6 h and ID1 levels were measured by western blots. **(F)** Hep3B Control and HIF1α-KD cells were cultured in normoxia or hypoxia overnight, and ID1 was IP and IB for the association with APC/CDH1. **(G)** PC3 cells were cultured in hypoxia with MG132 for 6 h. HIF1α was then IP and IB for associations with APC/CDH1 and ID1-ID4. **(H)** PC3 cells were cultured in hypoxia with MG132 for 6 h, HIF2α was then IP and IB for the association with ID1.

To understand the mechanism for hypoxia to degrade ID1, we used our established RNAi methods to knockdown (KD) HIF1α, HIF2α, or non-target control (Geng et al., 2011, 2012, 2018). We found that hypoxia reduced ID1 and ID3 among the ID-family proteins (Figure 2A). Inhibiting HIF1α rescued and reinstated ID1, but not ID3 (Figure 2A). On the other hand, inhibiting HIF2α had no effect (Figure 2B), suggesting ID1 protein is negatively affected by HIF1α, but not HIF2α. Further, we treated cancer cells with a sub-toxic dose of a pharmacological HIF inhibitor-digoxin (Zhang et al., 2008). We found that cells treated with digoxin consistently expressed higher levels of ID1 proteins in hypoxia compared to solvent-treated controls (Figure 2C). Most commonly, ID1 protein degradation is mediated by polyubiquitination (Bounpheng et al., 1999; Berse et al., 2004; Trausch-Azar et al., 2004). We found that ID1 polyubiquitination was significantly increased in hypoxia, and HIF1α was required for the increase (Figure 2D). Since HIF1α lacks the function of polyubiquitination, we determined the E3 ubiquitin ligase responsible for ubiquitination of ID1 in hypoxia. There are two known ID1 E3 ligases, Smurf2 and APC/CDH1 (Lasorella et al., 2006; Kong et al., 2011). We used siRNA to specifically inhibit Smurf2 and CDH1 as well as the HIF1α-E3 ligase VHL. We found that silencing APC/CDH1 significantly reinstated ID1 levels in hypoxia (Figure 2E), suggesting that HIF1α accelerates ID1 degradation in hypoxia through APC/CDH1. This possibility was further supported by results of protein co-IP experiments, in which we found that the ID1 and APC/CDH1 interaction was enhanced in hypoxia in HIF1α-expressing cells but not in HIF1α-KD cells (Figure 2F); in addition, HIF1α was able to interact with both ID1 and APC/CDH1 in hypoxia, but not with other members of the ID family (Figure 2G). In contrast, HIF2α was not found to be associated with ID1 (Figure 2H). Thus, it appears that there is a specific negative regulation of ID1 by HIF1α *via* APC/CDH1.

### ID1 Confers Resistance to HIF1α-Targeted Inhibitions

**FIGURE 3:**
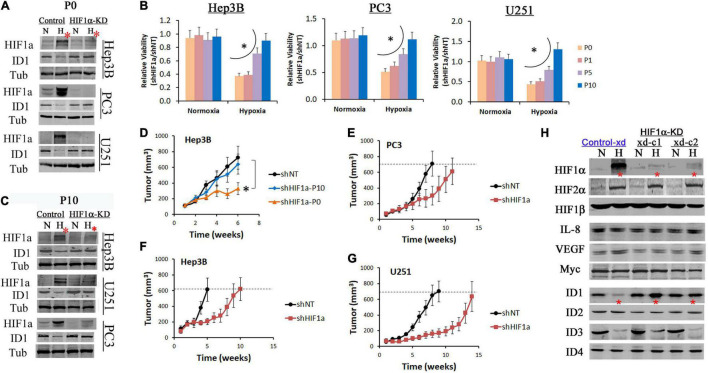
ID1 is upregulated in tumors resistant to HIF1α inhibition. Hep3B, PC3, U251 cells were stably transfected by lentiviral shRNA targeting HIF1α mRNA (HIF1α-KD) or non-specific (Control). The resulting cells were cultured in 20% O_2_ (N) or 1% O_2_ (H) for 24 h. **(A)** Western blots of HIF1α and ID1. Tubulin was used as loading control. The P0 indicates cells did not undergo serial passages in hypoxia. **(B)** Control and HIF1α-KD cells from Hep3B, PC3, and U251 underwent serial passages (1–10) in 20% or 1% O_2_. Cell viability was determined by viable cell numbers (shHIF1α/shNT) after P0, P1, P5 and P10. ^∗^*P* < 0.05, ANOVA, mean and stdv, *n* = 3. **(C)** Western blots of HIF1α and ID1 after P10. **(D)** Control (shNT) and HIF1α-KD cells (P0 and P10) from Hep3B were injected (3 million per injection) into the flank of male nude mice. Tumors were measured by digital caliper, ^∗^*P* < 0.05, *t*-test, mean and standard error of mean (SEM), *n* = 8. **(E–G)** Control and HIF1α-KD cells (P0) from PC3, U251, and Hep3B were injected (3 million per injection) into the flank of male nude mice. Tumors were measured by digital caliper, mean and SEM. **(H)** Hep3B-xd cells from were cultured in 20% or 1% O_2_ overnight. Protein levels were determined by western blot.

HIF1α is a drug target for anticancer therapies (Semenza, 2003). Since ID1 is a well-established oncoprotein that promotes cancer cell survival and proliferation (Perk et al., 2005), we hypothesize that the increase of ID1 may confer adaptive resistance to HIF1α-targeted inhibition. In prostate, liver and brain cancer cell lines, ID1 was inhibited in hypoxia in HIF1α-expressing controls, but was consistently reinstated in HIF1α-KD cells (Figure 3A). Stable HIF1α shRNA knockdown (HIF1-KD) initially inhibited tumor cell survival and growth in hypoxia (P0 in Figure 3B). However, resistance was developed *via* serial passage (P0-P10 in Figure 3B). In P10 cells, western blots confirmed that HIF1α protein remained inhibited, while ID1 was reinstated (Figure 3C). In xenograft experiments, we found that tumors established with the resistant cells (HIF1α-KD-P10) grew as aggressively as the HIF1α-expressing controls, suggesting that the *in vitro* hypoxia selected resistance was sufficient to confer resistance *in vivo* (Figure 3D). On the other hand, tumors by cells sensitive to HIF1α-KD (P0) exhibited a slower growth pattern, but grew to similar sizes as the controls (Figures 3E–G), suggesting *de novo* resistance. After tumors were harvested, we isolated human epithelial tumor cells from the Hep3B xenograft and re-establish them as xenograft-derived subclones (2 clones, Hep3B-xd-c1/2). Western blots showed that, in hypoxia, ID1 was inhibited in Control-xd cells, but was reinstated in HIF1α-KD-xd cells (^∗^ in Figure 3H). Within the ID family (ID1-ID4), ID3 was also inhibited in hypoxia, but not reinstated; ID2 and ID4 were unchanged (Figure 3H). Also, other proteins known to confer adaptation to HIF1 inhibitions, e.g., Myc, IL-8, and VEGFA, were unchanged (Figure 3H).

**FIGURE 4:**
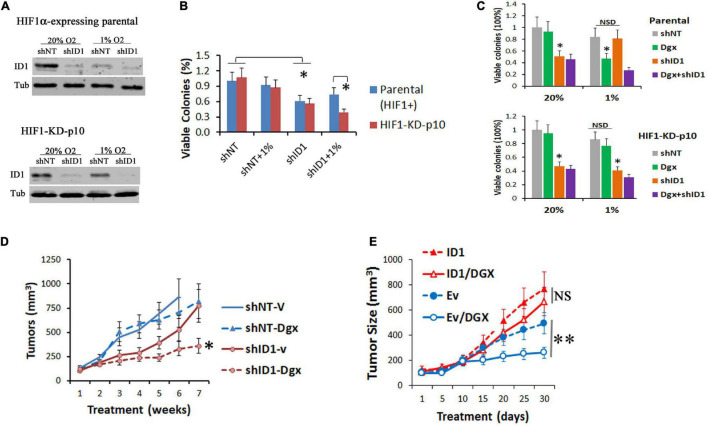
**(A)** Hep3B-parental and -HIF1α-KD-P10 cells were transduced with lentiviral shRNA-ID1. Cells were cultured in 20% or 1% O_2_ for 24 h, and ID1 and tubulin proteins were determined by western blots. **(B)** Cells in **(A)** underwent colony formation assays in 20% or 1% O_2_, ^∗^*P* < 0.05, *t*-test, mean and stdv, *n* = 3. **(C)** Parental-shNT/shID1 cells or HIF1α-KD-P10-shNT/shID1 cells were treated with HIF inhibitor digoxin and underwent colony formation assays. ^∗^*P* < 0.05, *t*-test, mean and stdv, *n* = 3. **(D)** HIF1α-KD-P10-shNT/shID1 cells were injected into the flank of nude mice, and tumor-bearing mice were treated by vehicle or digoxin (Zhang et al., 2008). ^∗^*P* < 0.05, ANOVA, mean and SEM, *n* = 6. **(E)** LNCaP-Ev/ID1 cells were injected into the flank of male nude mice, and tumor-bearing mice were treated by vehicle or digoxin. ^∗∗^*P* < 0.05, *t*-test, mean and SEM, *n* = 6.

To determine the role of ID1 in conferring the resistance above, we transduced the parental and resistant shHIF1α-p10 subclones with lentiviral shRNA silencing ID1 (Figure 4A), and performed colony formation assays in 20% and 1% O_2_. In normoxia, ID1-shRNA alone was growth inhibitory to both cell lines (Figure 4B). In hypoxia, ID1-shRNA had no effect to HIF1α-expressing parental cells, but significantly inhibited the shHIF1α-p10 (Figure 4B). Next, we treated the cells with HIF inhibitor digoxin. In hypoxia, we found that digoxin (100 nM) inhibited the colony formation of HIF1α-expressing cells, but not the resistant shHIF1α-p10 cells (Figure 4C). Importantly, ID1-shRNA was growth-inhibitory in hypoxia against the digoxin-resistant cells (Figure 4C). In xenograft experiments, HIF1α-KD-p10 tumors were resistant to digoxin, but stable ID1-shRNA increased the sensitivity (Figure 4D). On the other hand, ID1-low LNCaP xenografts were sensitive to digoxin, but not in ID1-overexpressing counterpart (Figure 4E).

### ID1 Confers HIF1-Independence *via* Metabolic Compensations

**FIGURE 5:**
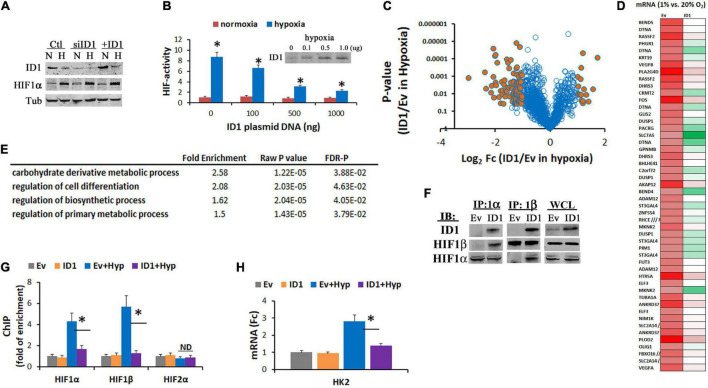
**(A)** Western blots of ID1, HIF1α, and tubulin (control) in Hep3B cells with siNT/siID1 or Ev/ID1-overexpression in 20% or 1% O2. **(B)** Hep3B-c1 cells that stably transfected with HIF-reporter system (hypoxia/HIF-driving firefly luciferase with constitutive renilla luciferase as control) were transfected with increasing amounts of ID1-overexpressing plasmids, and cultured in 20% or 1% O_2_ for 24 h. HIF-activity was determined by dual-luciferase measurements as described before (Zhang et al., 2008). ^∗^*P* < 0.05, ANOVA, mean and stdv, *n* = 3. Inserts: ID1 western blots. **(C)** LNCaP-Ev and LNCaP-ID1 cells were cultured in 20% and 1% O2 for 24 h, and then underwent Affymetrix cDNA microarray. Volcano plot of hypoxia-response genes in LNCaP-ID1 vs. LNCaP-Ev cells. **(D)** Heatmap of hypoxia-upregulated genes in Ev-cells, which were attenuated in ID1-cells, color: green → red = gene expressions low → high. **(E)** GO pathway analysis of hypoxia-upregulated genes that were attenuated by ID1. **(F)** Ev or ID1 cells were cultured in 1% O_2_ for 24 h. Whole cell lysates (WCL) were immunoprecipitated with antibodies for HIF1α, and probed for ID1, HIF1α, by western blots. **(G,H)** LNCaP-Ev or -ID1 cells were cultured in 20% or 1% O2 for 24 h. The enrichments of HIF1α, HIF1β, or HIF2α (relative to IgG control) at HK2 gene promoter were determined by ChIP **(G)**. The levels of HK2 gene expression were determined by qRT-PCR **(H)**. ^∗^*P* < 0.05, *t*-test, mean and stdv, *n* = 3.

HIF1α orchestrates hypoxia response by the upregulation of gene expressions. To further understand the interaction between HIF1α and ID1, we evaluated the effect of ID1 on HIF1α protein level and transcriptional activity. We found that ectopic increase or siRNA silencing of ID1 had no significant effect to HIF1α protein levels (Figure 5A), but attenuated its transactivation activity (ID1 transfection) (Figure 5B). We next used Affymetrix cDNA microarray to determine the effect of ID1 on hypoxia-induced gene expression (Figure 5C). We found that a subset of hypoxia-upregulated genes was significantly reduced in cells with ID1 overexpression (Figure 5D), among which genes mediating *biosynthesis*, *carbohydrate metabolism*, and *cell differentiation* were significantly enriched (Figure 5E). Mechanistically, we found that ID1 negatively regulates hypoxia-response genes by binding to HIF1 proteins (Figure 5F), and reducing HIF1 recruitments to the target gene promoter, e.g., HK2 (Figure 5G), and gene expression (Figure 5H).

**FIGURE 6:**
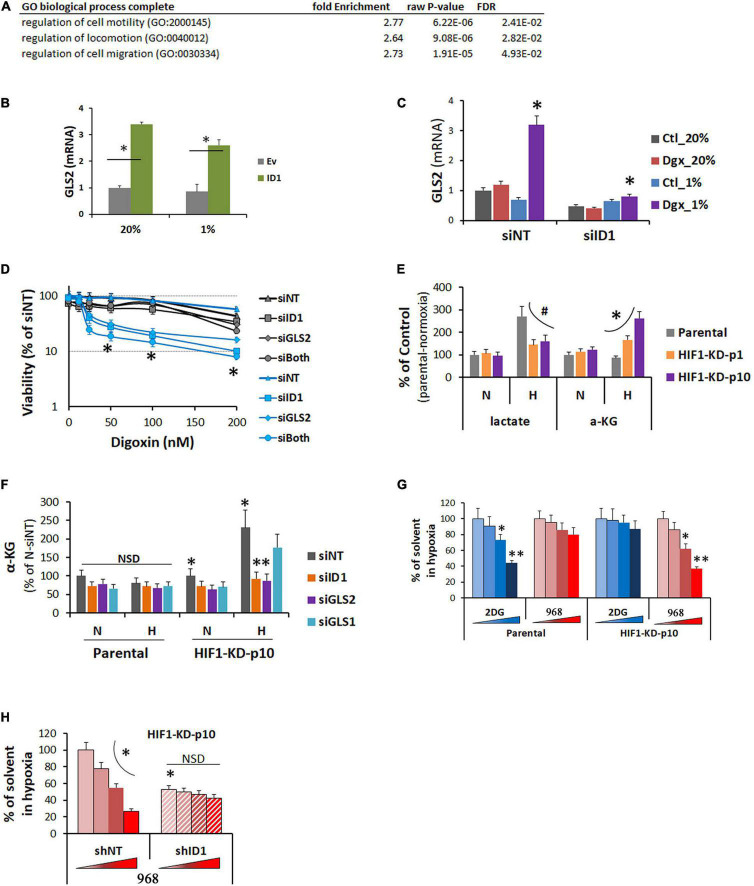
**(A)** GO pathway analysis of genes upregulated in LNCaP-ID1 vs. LNCaP-Ev cells. **(B)** LNCaP-ID1/Ev cells were cultured in 20% or 1% O_2_, and GLS2 mRNA were determined by cDNA microarray. ^∗^*P* < 0.05, *t*-test, mean and stdv, *n* = 3. **(C)** Hep3B parental cells were transfected with siRNA (siID1/siNT), and treated by digoxin in 20% or 1% O2 overnight. GLS2 were determined by qRT-PCR. ^∗^*P* < 0.05, *t*-test, mean and stdv, *n* = 3. **(D)** Hep3B-HIF1-KD-p10 cells were transfected with siRNA (siNT, siID1, siGLS2, or siID1++siGLS2), and treated by increasing concentrations of digoxin in 20% or 1% O2 for 72 h. Cell viability were determined by XTT. ^∗^*P* < 0.05, ANOVA, mean and stdv, *n* = 3. **(E)** Hep3B-parental, -HIF1α-KD-P1, or -HIF1α-KD-P10 cells were cultured in 20% or 1% O_2_ for 24 h. Levels of lactate and a-ketoglutarate (aKG) were determined by colorimetric assays from BioVision. #, ^∗^*P* < 0.05, ANOVA, mean and stdv, *n* = 3. **(F)** Hep3B-parental/HIF1α-KD-P10 cells were transfected with siRNA (siNT, siID1, siGLS1, or siGLS2), and cultured in 20% or 1% O2 overnight. Then, αKG was determined by colorimetric assays from BioVision. ^∗^, ^∗∗^*P* < 0.05, *t*-test, mean and stdv, *n* = 3. **(G)** Hep3B-parental/HIF1α-KD-P10 cells were treated with increasing doses of glycolysis inhibitor 2DG or GLS2 inhibitor 968 (Sigma) in 1% O_2_. Cell viability was determined by XTT. ^∗^, ^∗∗^*P* < 0.05, *t*-test, mean and stdv, *n* = 3. **(H)** HIF1α-KD-p10 cells were transfected with shNT or shID1, and treated by GLS2 inhibitor 968 in 1% O_2_ as in **(G)**. Cell viability was determined by XTT, ^∗^*P* < 0.05, *t*-test, mean and stdv, *n* = 3.

On the other hand, ID1 overexpression also increased gene expression. GO-term analysis showed that the increase was enriched at pathways for cell mobility and invasion (Figure 6A), in agreement with previous finding that ID1 promotes tumor cell migration and Matrigel invasion (Lyden et al., 1999). For the first time, importantly, we found that gene encoding for glutamine pathway enzyme GLS2 was significantly increased in ID1-overexpressing cells in normoxia and hypoxia (Figure 6B). In response to HIF inhibitor-digoxin, GLS2 was increased in ID1-dependent manner (Figure 6C). Functionally, we found that GLS2 played a role in mediating the ID1-dependent resistance to HIF1 inhibition. Cells that were resistant to digoxin were highly sensitive to siRNA targeting ID1 and GLS2 (Figure 6D). The adaptive response was associated with a switch in energy metabolism. In response to hypoxia, parental cells increased cytosolic glycolysis leading to lactate [the Warburg effect (Dang, 2007)] (Figure 6E). However, cells with stable HIF1α-KD did not have such response (# in Figure 6E). Instead, they exhibited an increase in glutamine metabolism (^∗^ in Figure 6E), which was sensitive to ID1 or GLS2 siRNA, but not affected by GLS1 siRNA (Figure 6F). To further understand the functional significance, we treated the parental and P10-resistant cells with inhibitors targeting glycolysis and glutamine pathways. We found that the parental tumor cells were more sensitive to glycolysis inhibitors in hypoxia (Figure 6G). As it developed adaptive resistance to HIF1α-KD, the sensitivity to glycolysis inhibitors diminished, while it became dependent to glutamine pathway and thus sensitive to glutamine pathway inhibitors (Figure 6G). Further, the switch of sensitivity to glutamine pathway inhibitors were negated by ID1-shRNA (Figure 6H), confirming the ID1-dependency.

## Discussion

Hypoxia is a common feature in solid tumors (Bhandari et al., 2019). The oncogenic role of ID1 in hypoxia is less characterized. By studying the interaction between ID1 and HIF1α, we here present a molecular model in which ID1 and HIF1α may drive tumorigenesis in non-hypoxic and hypoxic conditions, respectively. Cancer cells may use this interaction to become more adaptable to the pathologically variable oxygen conditions, thereby gaining survival and growth advantages. In terms of metabolisms, the ID1/HIF1-interaction explains why tumor cells use different energy substrates in non-hypoxic and hypoxic conditions. In non-hypoxic conditions, ID1 supports tumor cells by upregulating GLS2 and glutamine metabolism. In hypoxia, ID1 protein degradation is accelerated, while HIF1α is activated to support tumors by glycolytic genes and glucose metabolism. In terms of cell cycle regulation, ID1 is a well-established driver for cell cycle progression and proliferation in non-hypoxic condition (Sikder et al., 2003). In hypoxia, however, tumor cell may slow down the cell cycle to become more adaptable to the reduced oxygen. Therefore, HIF1α accelerates the protein degradation of ID1 *via* APC/CDH1, which is known to coordinate cell cycle progression by protein degradations (Sudo et al., 2001).

Due to the universal occurrence of hypoxia and the intratumor heterogeneity (Movsas et al., 1999; Bristow et al., 2014; Lalonde et al., 2014; Patel et al., 2014), the ID1/HIF1α-interaction may present challenge to HIF-targeted therapies. This may in part explain why HIF-targeted inhibitors have not shown sufficient efficacy in blocking hypoxic tumors. Based on our data, we expect that ID1 is increased in response to HIF-targeted inhibitions, and in turn plays a compensatory role supporting the survival, proliferation, and invasion of tumor cells in hypoxia. Therefore, silencing ID1 may restore or increase tumor sensitivity to HIF1α inhibition. It also provides a molecular basis for ID1 to be used as a biomarker to predict resistance or efficacy of HIF-inhibitors. In xenograft samples (Figure 3), we detected an increase of ID1 protein level by immunohistochemistry. However, we were unable to clearly define hypoxic regions of the tumor, due to the genetic or chemical inhibition of HIF1α, which we normally use to define hypoxia. Thus, the potential use of ID1 as a marker to predict HIF inhibitor efficacy requires the development of reliable markers of tumor hypoxia, other than HIF1α.

Tumor growth is driven by multiple oncogenic pathways (Hinohara and Polyak, 2019). Our study underscores the importance of understanding the interaction among them. The interaction between ID1/HIF1α revealed by us suggests a new level of complexity. Clinical tumors are heterogenic in oxygenation. However, most of the genomic analysis of clinical tumors does not differentiate cells based on oxygen. Signals from hypoxic cells can be diluted or masked by non-hypoxic cells. Therefore, the clinical validation of our model requires new approaches focusing on the hypoxic vs. non-hypoxic subsets, e.g., *via* single-cell analysis. We also speculate that the level of ID1 will be inversely associated with HIF1α in clinical tumor samples. Many types of tumors acquire HIF1α in normoxia by oncogenic mutations, thus the negative regulation of ID1 by HIF1α can occur in normoxia if HIF1α is available.

### Experimental Methods and Procedures

#### Cell Culture Conditions

Prostate (PC3, LNCaP), liver (Hep3B) and brain (U251) cancer cell lines were purchased from ATCC and cultured in RPMI or DMEM media with 10% FBS and 1% penicillin streptomycin. The hypoxia or 1% oxygen condition was created in the cell culture incubator by replacing oxygen with liquid nitrogen. Sodium Bicarbonate (30 mM) was used to neutralize the hypoxia-induced lactate acid for experiments without involving metabolic measurement.

#### cDNA Microarray

Gene expression profiles by Affimetrix cDNA microarray were determined at OHSU Gene Profiling Shared Resources as described before (Geng et al., 2018). Each condition had a biological triplicate (*n* = 3). False discovery (FDR) adjusted *t*-test was used to determine the differential expression of individual genes. The level of significance (differentially expressed) was set at Δlog2 expression > 1 or < −1 with the FDR-adjust *P*-value (*q*) < 0.05. The cDNA microarray data was also analyzed by Gene Set Enrichment Analysis (GSEA), and the enrichment of cancer hallmark pathways was determined with FDR-q.

#### Gene Knockdown

As previously described (Geng et al., 2010, 2018; Suwaki et al., 2011), pooled siRNA or lentiviral-based shRNA were purchased from Sigma, and carried out to silence genes, e.g., HIF1α, HIF2α, ID1, GLS1, and GLS2. Efficacies of siRNA and shRNA were all determined by western blots.

#### PCR, Western Blotting, ChIP-PCR

Quantitative PCR, ChIP-PCR and western blotting were done as previously described (Geng et al., 2018). All RT-PCR primers were purchased from Real Time Primers LLC, and have been verified for human RT-PCR. The antibodies for western blots were purchased from Abcam, BioCheck, R&D Systems, and Santa Cruz Biotechnology. The ChIP value was adjusted to the IgG as negative control.

#### Viability/Proliferation/Metabolic Analysis

Cell viability and proliferation were determined by colony formation assay, XTT, and/or trypan blue exclusion. Cellular lactate and a-ketoglutarate levels were determined by colorimetric kits from BioVision. All these experiments were done as previously described (Geng et al., 2010, 2011, 2018; Liu et al., 2013, 2015).

#### Xenograft Experiments

Subcutaneous implants of PC3, Hep3B, U251, and LNCaP cells, including HIF1, ID1 shRNA or overexpressing subclones, were generated in male nude mice as previously described (Geng et al., 2018). The tumor volume was determined by digital caliper measurement, and expressed as % of growth relative to the start of treatment. All animal experiments are in compliance with protocols approved by OHSU and Johns Hopkins IACUC.

#### Hypoxia Inducible Factor-Activity Reporter Assays

As previously described (Zhang et al., 2008), plasmids encoding the firefly luciferase reporter gene under the control of hypoxia response elements or the constitutive renilla luciferase gene (gifts from Dr. Gregg Semenza at the Johns Hopkins University) were co-transfected into the cells, and dual luciferase reporter gene assays were performed with the kit from Promega.

#### Statistical Analysis

All experimental data were expressed as mean and standard deviation (SD) unless indicated otherwise. Statistical comparisons between two sample sets were performed with student *t*-test or paired *t*-test, comparisons among more than two samples were performed with repeated measures ANOVA, using MedCalc software. *P* < 0.05 was considered as significant, and *P* ≥ 0.05 was considered as not significant different (NSD).

## Data Availability Statement

The cDNA microarray data is deposited at Gene Expression Omnibus, GSE185563.

## Ethics Statement

The animal study was reviewed and approved by the Oregon Health & Science University and the Johns Hopkins University, Institutional Animal Care and Use Committee.

## Author Contributions

DQ and SK designed the study. DQ, SK, and HG analyzed the data and wrote the manuscript. TB, SK, and H-KK provided research tools and methods. HG, CH, JP, CX, QL, SW, and SK performed the experiments. All authors contributed to the article and approved the submitted version.

## Conflict of Interest

The authors declare that the research was conducted in the absence of any commercial or financial relationships that could be construed as a potential conflict of interest.

## Publisher’s Note

All claims expressed in this article are solely those of the authors and do not necessarily represent those of their affiliated organizations, or those of the publisher, the editors and the reviewers. Any product that may be evaluated in this article, or claim that may be made by its manufacturer, is not guaranteed or endorsed by the publisher.
